# Clinical outcome of curettage in atypical cartilaginous tumors of the long bones: a descriptive cohort study

**DOI:** 10.2340/17453674.2024.42636

**Published:** 2024-12-23

**Authors:** Claire H J SCHOLTE, Michiel A J VAN DE SANDE, Robert J P VAN DER WAL, Demien BROEKHUIS, Kirsten VAN LANGEVELDE, Desirée M J DORLEIJN

**Affiliations:** 1 Department of Orthopedics, Leiden University Medical Centre, Leiden, The Netherlands; 2 Department of Radiology, Leiden University Medical Centre, Leiden, The Netherlands; 3 Department of Orthopedics, University Hospital Ghent, Ghent, Belgium

## Abstract

**Background and purpose:**

Despite evolving management strategies for atypical cartilaginous tumors (ACT)—shifting from radical resection to intralesional curettage and “wait-and-scan” approaches—there remains no universal consensus on optimal treatment. We primarily aimed to evaluate disease-specific and progression-free survival following intralesional curettage and adjuvant phenol treatment of ACTs. Secondary aims included assessing surgical complications, the need for additional interventions, and an overview of long-term follow-up.

**Methods:**

This retrospective cohort study of 388 ACT patients was conducted at a tertiary referral center from 2000 to 2019. Comprehensive data collection included demographics, tumor characteristics, and follow-up outcomes.

**Results:**

Residual disease was observed in 14% (n = 53) of cases, with continued growth on sequential imaging in one-fourth (n = 13 of 53). Postoperative fractures occurred in 10% (n = 37) after a mean of 7 months, and 16% (n = 61) required a second surgery due to pain or joint movement limitations. There was no malignant progression or mortality observed.

**Conclusion:**

We found that curettage for ACT is not associated with mortality or malignant progression but does carry risks of complications and residual disease. This raises important questions regarding the necessity of surgical intervention. Further research is needed to refine the treatment approach for ACT.

The World Health Organization (WHO) reclassified chondrosarcoma grade I as “Atypical Cartilaginous Tumour (ACT)” in 2013, further refining the terminology in the 2020 updated classification to distinguish ACT for the extremities and chondrosarcoma (CS) grade I for lesions in the axial skeleton. Histologically, ACT shows local aggressiveness and low cellularity. These tumors do not metastasize [1-2]. In recent years, the incidence of ACTs has shown a threefold increase from 2.88 per million per year (1989–1996) to 8.78 per million per year (2005–2013) [3]. However, no change was observed in the incidence of high-grade chondrosarcoma (HGCS). This surge in ACTs can be attributed mostly to the expanded use of magnetic resonance imaging (MRI) examinations) [3]. Before 2013 ACTs were classified as grade I (low grade malignant) chondroid tumors, characterized by their local aggressiveness, whilst grades II and III were considered high-grade malignant [4]. The majority of grade I tumors emerge de novo, with a small percentage originating from pre-existing enchondromas [5]. Classification is based on histological and MRI features, with ACT primarily diagnosed on MRI despite the ongoing debate regarding the necessity of preoperative biopsies for histological evaluation [6-8].

Management of ACT has evolved significantly, transitioning from radical resection to intralesional curettage or a wait-and-scan strategy involving periodic MRI surveillance. 2 previous studies have shown promising results for the wait-and-scan approach and emphasize the absence of progression to HGCS [9-16]. However, there is no long-term follow-up data available yet for this wait-and-scan strategy. To address this limitation, we implemented a 5-year follow-up period in this study, as opposed to the commonly recommended 3- to 4-year follow-up [17]. Given the limited long-term follow-up data for patients undergoing intralesional curettage for ACT, we opted for a longer and more conservative approach. This extended follow-up allows for better monitoring of potential late recurrences or complications, ensuring that we gather adequate data to inform clinical practice and improve patient outcomes.

Notably, there is no universal consensus on the preferred treatment strategy for intra-osseous cartilaginous tumors, leading to variation in treatment approach worldwide [9,10,18,19]. The WHO’s 2013 reclassification of grade I CS as ACT significantly influenced the shift towards less aggressive treatment strategies for managing these tumors. However, studies by Dierselhuis et al. [20] and Deckers et al. [9] have questioned the effectiveness of curettage in preventing the transformation of ACT into HGCS and highlighted post-curettage complications and residual tumor and recurrence of tumor [9-11,18-20].

We primarily aimed to evaluate disease-specific and progression-free survival following intralesional curettage and adjuvant phenol treatment of intra-osseous cartilaginous tumors in a tertiary referral center. Secondary aims include assessing surgical complications and the need for additional interventions.

## Methods

### Study design

This retrospective study, conducted at a tertiary referral center for bone and soft-tissue tumors, focused on patients undergoing intralesional curettage for an intra-osseous cartilaginous tumor occurring in the long bones from 2000 to 20^19^, with a histological proven ACT according to the WHO guidelines and a minimum of 2-year follow-up with MRI after surgery [1]. Exclusion criteria were radiologically or histologically diagnosed enchondromas, and patients with Ollier’s disease or Maffucci syndrome.

The study was reported according to STROBE guidelines.

### Radiological evaluation

A specialized musculoskeletal (MSK) radiologist re-evaluated the sequential MRI scans in cases where the radiological reports were unclear regarding recurrent or residual disease. Therefore, residual disease was defined as the presence of cartilage nodules surrounding the curettage cavity on the first MRI conducted within 1-year post-surgery. Radiologically undetectable cartilage nodules on the first postoperative MRI that became visible on a sequential postoperative MRI were defined as recurrence.

### Data collection

Patients’ records were examined for data on mortality, demographics, tumor characteristics, imaging methods, recurrences, residual tumors, surgical details, and follow-up. Patients were identified through the hospital coding registry. Lesion size was determined by the longest diameter given in the radiology report.

### Treatment and follow-up

Between 2000 and 2013 all patients with an ACT diagnosed on MRI were treated with curettage, phenolization, and cancellous bone chips. After 2013, treatment decisions varied between curettage and wait-and-scan [1]. Surgical treatment involved curettage, with adjuvant therapy (phenol). Allograft bone chips or void filling such as cement and prophylactic plate fixation were applied depending on size and location and according to the surgeon’s assessment. MRI was implemented for postoperative follow-up, differentiating residual tumors from recurrences at 1, 3, and 5 years after curettage.

Residual lesions and residual growth were diagnosed and followed up by interval MRI. If a biopsy was performed before curettage, this histological diagnosis was collected. The histological diagnosis of the curettage tissue was collected and compared with the biopsy diagnosis. The histological diagnosis of an ACT was defined as a lobulated growth pattern with abundant hyaline cartilage matrix and low cellularity. The tumor lobules usually permeate and entrap the pre-existing lamellar bone trabeculae and mitoses are absent. Necrosis may be present [2].

### Complications and secondary interventions

Data on complications (e.g., fractures, infections) and second interventions or treatment such as radiofrequency ablation (RFA), second curettage, or surgical complication management was collected. The frequency of these second interventions over time was evaluated.

### Statistics

IBM SPSS Statistics (v. 25; IBM Corp, Armonk, NY, USA) facilitated data analyses. Descriptive statistics were calculated for the length of time to events. In the case of non-normal distribution of the data, the median was used as the measure of central tendency, providing a more accurate representation of the central point in the dataset. A chi-square test was performed to assess the association between tumor size and the presence of residual tumors, categorizing tumors as < 50 mm or ≥ 50 mm. P < 0.05 was considered statistically significant.

### Ethics, registration, data sharing, use of AI, funding, and disclosures

Ethical approval was obtained from Medical Ethical Review Board of Leiden, The Hague, and Delft (METC LDD), number G21.160. This study was registered with the Department of Orthopedics at the Leiden University Medical Center (LUMC). The data supporting the findings of this study is not publicly available due to privacy considerations. No artificial intelligence or automated decision-making tools were utilized in the analysis or interpretation of the data. No specific funding was received for this study. The authors declare no conflicts of interest related to this study. Complete disclosure of interest forms according to ICMJE are available on the article page, doi: 10.2340/17453674.2024.42636

## Results

### Patients

**Figure 1:**
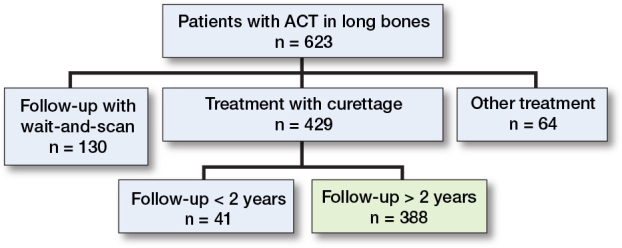
Flow diagram of the study participants.

623 patients with an ACT in the long bones underwent screening for eligibility (Figure 1). 64 underwent other treatment such as RFA or en-bloc resection and 130 were followed-up through wait-and-scan. The last group was predominantly found over the last 5 years of this study, as the treatment paradigm shifted towards a wait-and-scan policy as preferred treatment within our center. RFA was used only briefly in relatively small lesions (< 2.5 cm). Resection of ACT lesions was performed in eccentrically located metaphyseal lesions. This left a total of 429 patients, of whom 41 had follow-up of less than 2 years due to being lost to follow-up or because 2 years had not yet passed since surgery. The final analysis therefore focused on 388 patients. The complete flowchart (including patients with wait-and-scan treatment) of the study has been published previously [16].

### Baseline characteristics of selected patients

**Figure 2:**
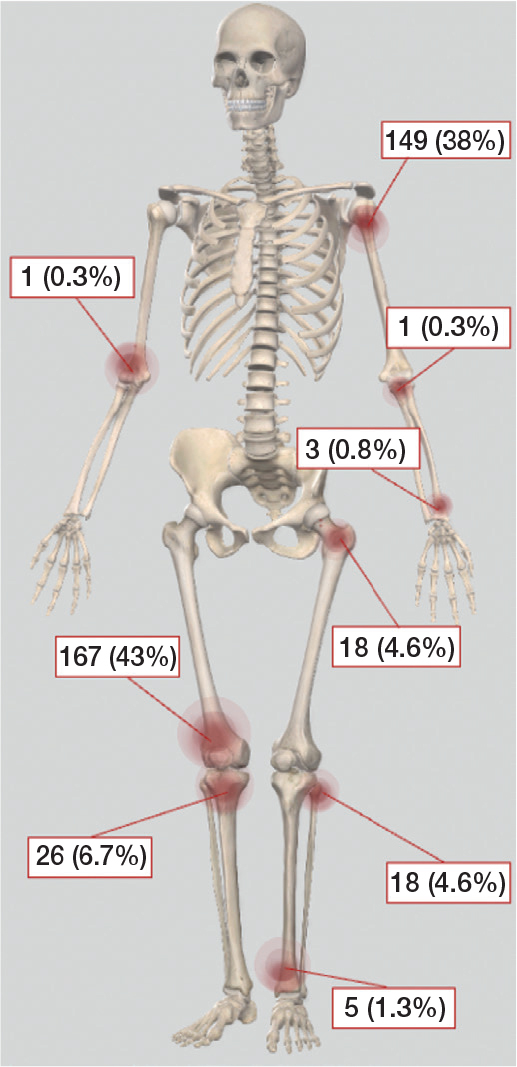
Skeletal distribution of 388 patients with ACT treated with intralesional curettage.

Baseline characteristics of the 388 patients showed 127 (33%) male patients, and a mean age of 50 years (Table). Most of the ACTs were in the proximal humerus or distal femur (Figure 2).

**Table:** Patients’ characteristics at baseline. Values are count (%) unless otherwise specified

Total	388
Male	127 (33)
Age, mean (SD)	50 (11)
ACT size in mm	
≤ 20	30 (7.7)
21–50	196 (51)
51–100	150 (39)
101–150	10 (2.6)
> 150	2 (0.5)
Preoperative histology needle biopsy **^a^**	87 (22)

SD = standard deviation. ACT = atypical cartilaginous tumor.

a In all instances, the ACT biopsy and post-curettage pathology reports consistently show concordant findings and, in all cases, ACT was diagnosed.

**Figure 3:**
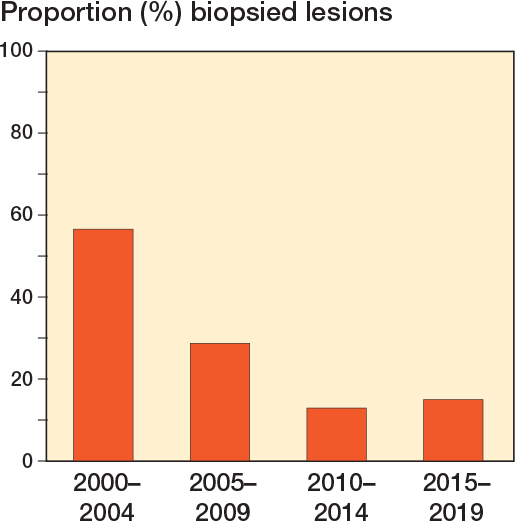
Histogram of biopsy utilization in 388 histologically proven ACT patients treated with intralesional curettage over time.

Whilst the overall utilization of MRI has increased in recent decades, the use of biopsy as a diagnostic tool at baseline has decreased in this cohort, as indicated in Figure 3. Notably, in earlier years, biopsy was more often performed as a work-up procedure to confirm the diagnosis. In more recent years, if MRI showed no aggressive characteristics suggesting an HGCS, histology tissue was obtained from the curettage procedure alone. In all instances, the ACT biopsy and post-curettage pathology reports showed concordant findings.

### Primary outcomes

**Figure 4:**
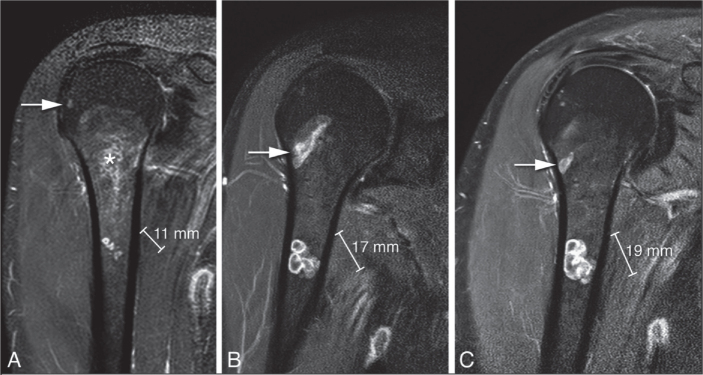
Coronal T1 fat-suppressed contrast-enhanced images of residual chondroid tumor nodules in the humerus that grew 8 mm in 9 years. The asterisk indicates the curettage cavity filling in with fatty bone marrow over time. The arrow points to a ganglion cyst.

**Figure 5:**
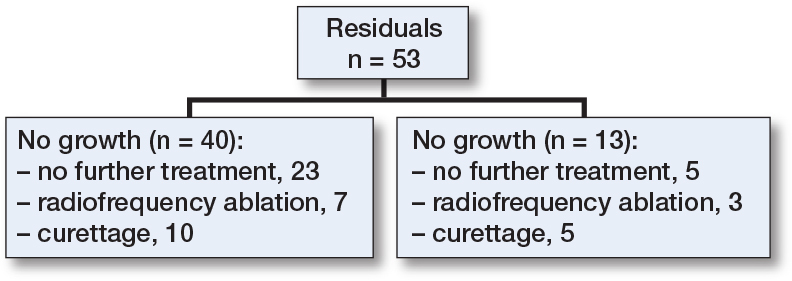
Flow diagram of residual follow-up. RFA = radiofrequency ablation.

**Figure 6:**
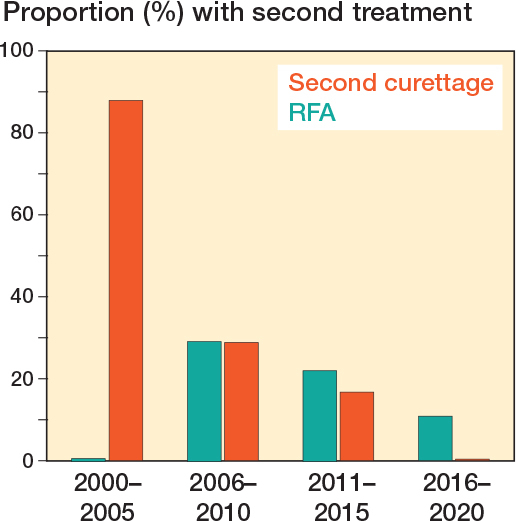
Histogram of the proportion of patients with RFA or second curettage performed for a residual per time period.

There was no disease-specific mortality observed related to the ACT. Residual disease was diagnosed in 53 patients (14%). Of these, 13 showed continuous growth on sequential imaging. The likelihood ratio for having a residual tumor was independent of ACT size (</≥ 50 mm). After residual disease was diagnosed, it was often monitored for 1 year, and if growth persisted it was addressed with either re-curettage (n = 5) or RFA (n = 3). Between 2006 and 2010, RFA exhibited a peak, yet subsequently demonstrated a decline over time. Figure 4 provides a clear example of such a growing residual tumor, illustrating an increase of 8.6 mm over 6 years. However, RFA or re-curettage was not consistently employed in the case of growth (Figure 5). At each RFA and re-curettage procedure, histologic material was collected, and all still showed evidence of ACT. A wait-and-scan follow-up was implemented in most cases. We showed a downward trend in the number of re-treatments (second curettage or RFA) over time (Figure 6).

### Secondary outcomes

Fractures following curettage occurred in 37 of the 388 patients (10%), with the highest incidence observed in the proximal humerus (8 fractures) and the distal femur (26 fractures). These locations accounted for 38% and 43% of all the ACT cases in the study, respectively. On average, fractures occurred after a median of 7 months post-diagnosis (IQR 4–16).

Amongst the 26 fractures in the distal femur, 3 patients had received prophylactic stabilization with a plate. In total, 36 patients had prophylactic plates. Despite the higher incidence of fractures in the proximal humerus and distal femur, no significant positive likelihood ratios were observed for any ACT location in relation to fracture occurrence. Of the 37 fractures, 11 were treated conservatively.

61 of the 388 patients (16%) required a second surgery after the initial curettage. Reasons for second surgery included pain and/or joint movement limitation due to, for example, complaints of preventive plate fixation (n = 12), fracture (n = 28), and/or other reasons such as patient’s anxiety or residual tumor. On average, second surgery was done at a median of 27 months after the first surgery (IQR 11–54).

## Discussion

We primarily aimed to evaluate disease-specific and progression-free survival following intralesional curettage and adjuvant phenol treatment of ACTs. No patient mortality or malignant progressions of tumors was observed. In this cohort study, we present the largest cohort of patients with ACT treated with intralesional curettage and found that the treatment is associated with a relatively high risk of residual tumor and treatment-related fractures, prompting a critical evaluation of its overall benefits.

The residual rate of 14% raises the question of why to perform intralesional curettage when there is a relatively high chance that micro- or macroscopic residual tumor will show growth over time.

**Figure 7:**
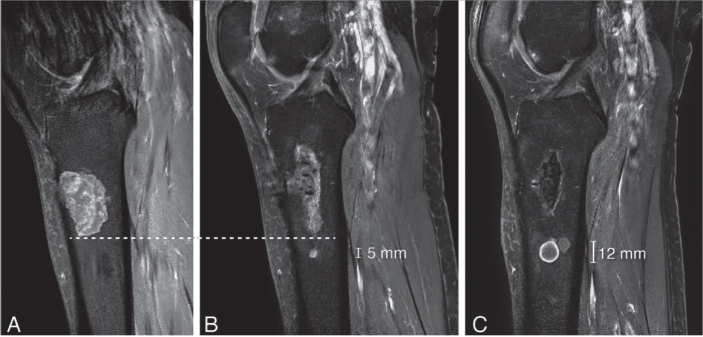
Sagittal T1 fat-suppressed contrast-enhanced images of a recurrence in the tibia that grew 7 mm in 8 years. (A) MRI at diagnosis showed a cartilage tumor in the proximal tibia diaphysis, in keeping with a histologically confirmed ACT after curettage. (B) A new cartilage nodule of 5 mm was noted 5 mm below the curettage cavity after 2 years. (C) The recurrence increased in size to 12 mm after 8 years. The patient remains in follow-up and has received no further treatment. Note the curettage cavity decreased in size over time and filled in with normal fatty marrow as a sign of healing.

A Cochrane review from 2019 reported a recurrence rate of ~5% [20]. In that study, no definition was provided for what was considered a recurrence or a residual tumor. In our study, of 388 patients, 1 patient (0.3%) was defined as a recurrence, which occurred 18 months after surgery. The recurrence was in the proximal tibia, demonstrating 7 mm growth over an 8-year period (Figure 7). No histological progression to malignancy was found in this recurrent case. After extensive review, this was the only patient who showed no abnormalities on the 1-year MRI but later developed a recurrent cartilage tumor. This finding suggests that the 1-year MRI can be considered a reliable standard for follow-up. It is important to highlight that we reassessed all cases with an unclear definition of recurrence versus residual disease to avoid misclassification. Some cases were initially categorized as recurrence in our study but were later identified as residual disease upon reassessment by a dedicated MSK sarcoma radiologist. This careful review process introduces a potential explanation for the observed disparity, indicating that diagnostic nuances may contribute to the lower recurrence rate reported in our study compared with the review by Dierselhuis et al. [20]. Consideration may be given to omit from further follow-up MRIs if the initial post-curettage MRI showed no residual tumor.

Our data demonstrated a trend towards a more conservative approach in both biopsy and second treatment over time. In our findings, there has been a gradual decline in the utilization of biopsies as a conventional diagnostic method over time. Notably, in earlier years, biopsy was more often performed as a work-up procedure to confirm the diagnosis. The same gradual decline applies for the performance of second curettage in the case of a residual tumor, especially over the past decade. This could explain why, in our study, no clear correlation can be identified between the growth of a residual tumor and the performance of a second curettage. In contrast to earlier practices where second curettage or RFA was often performed even in the absence of growth, contemporary approaches prioritize a more reserved stance, reflecting a nuanced shift in treatment strategies. In earlier cases, residual tumors were sometimes re-curetted up to 3 times, whereas current practice leans towards leaving a residual tumor in situ rather than pursuing repeated curettage.

A recently published study by Krebbekx et al. found an overall fracture risk of 6% [12]. Whilst the Cochrane review reported a complication rate of 4%, mainly fractures, our study revealed an notably even higher fracture incidence of 10%. This can be explained by several factors such as operation technique (type of cortical window) and the (dis)use of plate fixation. In our center in 2007, we transitioned from a square cortical bone window to an elliptical bone window alongside a more aggressive policy to use plates to reduce the potential risk of fractures. Future research will focus on the impact of treatment strategies to better understand their effectiveness in fracture prevention and potentially explain the higher fracture incidence observed in our study.

### Limitations

Some files retained incompleteness in treatment and follow-up variables. The changes in ACT definition in 2013 by the WHO led to inconsistent decision-making on whether to perform curettage, compounded by the unregistered reasons for these decisions, relying on the doctors’ and patients’ personal experience and beliefs. As a result, there is a potential for confounding by indication. Our data reflects (shared) clinical decision-making in a period when a shift towards less surgery in ACT was developed.

### Conclusion

We found that curettage for ACT is not associated with mortality or malignant progression but does carry risks of complications and residual disease. This raises important questions regarding the necessity of surgical intervention. Further research is needed to refine the treatment approach for ACT.

## Notes

Conceptualization: DMJD and MAJvdS; methodology: DMJD, KvL, and MAJvdS; formal analysis: CHJS; resources: MAJvdS; data curation: CHJS; writing original draft: CHJS; writing review and editing: DMJD, KvL, RJPvdW, DB, and MAJvdS; visualization: CHJS and KvL; supervision: DMJD, KvL, RJPvdW, and MAJvdS.

The authors thank G. Kracht for figure editing.

Handling co-editors: Ilkka Helenius and Robin Christensen

*Acta* thanks Minna K Laitinen and Pietro Ruggieri for help with peer review of this manuscript.
